# Repeated sampling improved the sensitivity of malaria microscopy in children under six years

**DOI:** 10.1186/s13104-020-05359-w

**Published:** 2020-11-04

**Authors:** Enoch Aninagyei

**Affiliations:** Department of Biomedical Sciences, School of Basic and Biomedical Sciences, University of Health and Allied Sciences, PMB 31, Ho, Volta Region Ghana

**Keywords:** Malaria microscopy, Repeated sampling, Baseline parasitaemia, Children up to 5 years, Suspected malaria patients

## Abstract

**Objective:**

Microscopy remains the gold standard for identification of malaria parasites. However, the sensitivity of malaria microscopy is low. This study evaluated the impact of repeated sampling up to 12 h in 177 children < 6 years with suspected malaria.

**Results:**

The median age was 3 years (interquartile range, 2.0–4.0 years). Eighty-nine percent (158/177) presented with hyperthermia together with one or more of the following symptoms: chills, headache, sweating, fatigue, nausea, abdominal pain, vomiting, diarrhea and cough. Baseline microscopy confirmed malaria in 29.9% (53/177) of the suspects. Repeated testing at 6 and 12 h increased the positive detection rates to 35.0% (62/177) and 41.8% (74/177), respectively. Microscopy underestimated malaria diagnosis by 11.9% on single testing. Children showing classical signs of malaria but with initial negative parasitological reports should be retested between 6 to 12 h to confirm or rule out a diagnosis of malaria.

## Introduction

Globally, human malaria is caused by five *Plasmodium* species, namely, *Plasmodium falciparum*, *P. vivax*, *P. malariae*, *P. ovale,* and *P. knowlesi* [1]. These parasites are responsible for over 228 million cases of malaria annually, resulting in about 405,000 deaths [2]. In sub-Saharan Africa, children under 5 years are at risk for malaria, and a significant number succumb to malaria if the diagnosis and treatment are delayed [3]. In malaria endemic countries, the number of malaria cases seems to be increasing, due to a decline in malaria control strategies which result in increasing transmission of parasites [4]. Key to malaria control is accurate detection of the parasites and treating with potent anti-malarials. Due to this, the World Health Organization (WHO) recommends parasitological confirmation of all suspected malaria cases before commencement of treatment [5].

Microscopy remains the gold standard in the laboratory identification of malaria [6]. However, the technique is unreliable in detection parasitaemia less than 500 parasites/μL of blood [7–9]. Despite being of low sensitivity, malaria microscopy could speciate *Plasmodium* parasites, and is useful in determining the degree of parasitaemia [10]. In cases of submicroscopic parasitaemia, microscopy is almost useless [11]. All *P. falciparum* infections start sub-microscopically before the detection limit is exceeded. In individuals with incompetent anti-malaria immunity such as children under 5 years, malaria-specific symptoms are usually provoked by low levels of parasitaemia. In this study, a strategy of repeated malaria testing of children up to 5 years presenting with suspected malaria was adopted, to evaluate whether smears repeated at 6 and 12 h after the initial sample improved the diagnostic yield.

## Materials and methods

### Study design, study site, and study population

An observational study was conducted at Ga West Municipal Hospital, Amasaman, in the Greater Accra Region, Ghana from March through December 2018. The study participants were children up to 5 years that were suspected of malaria. Parental written informed consent was obtained for serial finger prick samples at baseline, 6 h and 12 h. During admission, children with negative malaria smears did not receive parenteral fluids nor anti-malaria drugs. Once malaria was confirmed, anti-malarial chemotherapy was commenced immediately. Children that remained febrile received tepid sponging and antipyretic therapy. Guardians completed a questionnaire to elicit responses on malaria risk assessment.

### Definition of suspected malaria

Any child with axillary temperature above 37.5 °C or rectal temperature above 38.5 °C of any duration with or without any other clinical complaints was identified as a suspected malaria case. All suspected cases of malaria were identified by pediatricians. Data on all other clinical history and/or presenting complains were collected from the patients’ folders.

### Parasitological confirmation of malaria

Detection of *P. falciparum* specific histidine-rich protein 2 (Pfhrp2) was done with CareStart™, following the manufacturer's instructions. Finger prick blood samples (approximately 6 µL of whole blood) were used to prepare thick blood films which were air dried, stained with 10% Giemsa for 10 min, and examined using light microscopy to quantify parasitaemia according to the WHO protocol [12].

### Data analysis

The frequencies of demographic parameters, malaria risk assessment, and presenting complaints were presented as percentages. Detection rate for both microscopy and RDT were calculated based on the total of tested for that category. In subsequent testing, all cases previously confirmed as malaria were excluded. The differences in baseline, 6-h and 12-h parasitological assessment were determined by Chi-square. Significant differences were defined as p < 0.05. Statistical analysis was done by SPSS Version 24 (Chicago, IL, USA).

## Results

Blood samples were taken from 177 children with suspected malaria. The median age was 3 years (interquartile range (IQR), 2.0–4.0 years), and 61.0% (108/177) were male. Most of the guardians (54.8%) were self-employed. Most (61.6%) of the patients lived close to open drains. Although 83.6% households owned long-lasting insecticidal nets (LLIN), these were not regularly used in 61.5% of households. Insecticide repellents were used in 51.4% of households. A large proportion of children (63.8%) were nocturnally active, and 21.4% slept in rooms with eaves (Table 1).

**Table 1:** Demographic and malaria risk exposure parameters of the suspected patients

Parameters	Number of patients	%
Age (years)		
1	17	9.6
2	33	18.6
3	39	22.0
4	47	26.5
5	41	23.2
Gender		
Male	108	61.0
Female	69	39.0
Category of guardian’s occupation		
Unemployed	16	9.0
Self-employed	97	54.8
Regularized private sector	43	24.3
Civil servant	21	11.8
Living close to open drains		
Yes	109	61.6
No	68	38.4
LLIN availability		
Yes	148	83.6
No	29	16.4
Regular LLIN usage		
Yes	57	38.5
No	91	61.5
Insecticide repellant usage		
Yes	91	51.4
No	86	48.6
Staying in rooms with eaves		
Yes	38	21.4
No	139	78.5
Nocturnal outdoor activity		
Yes	113	63.8
No	64	36.2

*LLIN* long-lasting insecticide net

Table 2 represents the clinical history of the children on direct questioning and examination. Almost all the children were hyperthermic (98.3%). The majority (> 50%) also experienced chills, headache, sweating, fatigue, and nausea while < 50% reported abdominal pain, vomiting, diarrhea, cough or rash. 89.2% (158/177) presented with more than one symptom.

**Table 2:** Presenting history of suspected malaria patients on arrival and during follow-up

Clinical symptoms	On arrival	%
Fever	174	98.3
Chills	153	86.4
Headache	148	83.6
Sweating	133	75.1
Fatigue	131	74.0
Nausea	98	55.4
Abdominal pain	77	43.5
Vomiting	68	38.4
Diarrhea	63	35.6
Cough	61	34.5
Rashes	53	29.9

The detection rate of malaria in baseline blood samples was 29.9% (53/177) using microscopy and 42.9% (76/177) using RDT (*x*^2^ = 5.90; p = 0.015). At 6 h repeat sampling in children with initially negative results, 9 (5.1%) and 3 (1.7%) additional positive malaria cases were detected using smear and RDT, respectively. At 12 h repeat sampling in children with negative results at the baseline and 6 h testing time points, and additional 12 (6.8%) and 5 (2.8%) positive malaria cases were detected using smear and RDT, respectively. Serial microscopy testing increased the malaria detection rate by 11.9% compared to a single smear at baseline (from 29.9% to 41.8%) which was equivalent to the detection rates from a single RDT (42.9%) (Table 3). Serial RDT testing increased the detection rate by 4.6% (from 42.9% to 47.5%). At baseline, RDT was superior (p < 0.0001). However, testing 6 (p = 0.041) and 12 (p = 0.023) hours respectively after baseline, microscopy became better. Baseline parasitaemia range was 9815–83,452 parasites/µL of blood. Parasitaemia range drastically reduced at 6-h (1708–4416) and 12-h (2205–7110) retesting (Table 3).

**Table 3:** Baseline and follow-up detection of malaria parasites

Sampling time	Diagnostic methods				
	Microscopy		Malaria rapid test		
	Number of patients	%	Number of patients	%	McNemar's test
Baseline					21.0 (p < 0.0001)
Positive	53^a,b^	29.9	76^c,d^	42.9	
Range of parasitaemia (/µL)	9815–83,452				
Mean axillary temperature	38.3 °C				
Negative	124	70.1	101	52.5	
Mean axillary temperature	37.1 °C				
6 h re-sampling on baseline negative patients					4.1 (p = 0.041)
Positive	9	7.9	3	3.0	
Range of parasitaemia (/µL)	1708–4416				
Mean axillary temperature	37.7 °C				
Negative	105	92.1	98	97.0	
Mean axillary temperature	37.7 °C				
Cumulative positive patients	62^a^	35.0	79^c^	44.6	
12 h sampling on 12-h negative results					5.1 (p = 0.023)
Range of parasitaemia (/µL)	2205–7110				
Positive	12	11.4	5	5.1	
Mean axillary temperature	37.8 °C				
Negative	93	88.6	93	94.9	
Mean axillary temperature	37.6 °C				
Cumulative positive patients	74^b^	41.8	84^d^	47.5	
Total malaria negative samples	103	58.2	93	52.5	

^a^*x*^2^ statistic 1.04, p = 0.307

^b^*x*^2^ statistic 5.42, p = 0.019

^c^*x*^2^ statistic 0.10, p = 0.748

^d^*x*^2^ statistic 0.73, p = 0.392

## Discussion

Compared to microscopy, the sensitivity of rapid diagnostic test (RDT) kits is significantly higher. This is because RDT kits are manufactured to detect *Plasmodium* spp antigens in individuals with parasitaemia of 50 to –100 parasites/µL of blood [7]. Even though RDT kits are very useful in malaria diagnosis, they are unable to determine parasite densities as well as treatment success or failure. Additionally, considering increasing prevalence of Pfhrp2/Pfhrp3 mutant *P. falciparum* in Ghana [13] and elsewhere [14], malaria microscopy becomes extremely useful in their detection. In view of this, microscopy remains essential in malaria management. Several studies have confirmed the lower sensitivity associated with malaria microscopy [7–9]. However, to be able to monitor malaria treatment success, determination of baseline malaria parasitemia prior to treatment is essential.

In this study, malaria microscopy at baseline detected malaria parasites in 30% of children up to 5 years that presented with a clinical suspicion of malaria. Repeat smears, conducted at 6 and 12 h after admission increased the diagnostic yield considerably, with an additional 21 (11.9%) children diagnosed smear positive. Even though tepid sponging and the use of antipyretics were expected to significantly reduce body temperature within an hour [15], axillary temperature of 37.7–37.8 °C was obtained for the additional patients in which malaria parasite were confirmed. Strikingly, the cumulative detection rate of malaria microscopy up to 12 h of initial negative parasitological results was very comparable to the detection rate of baseline RDT (*x*^2^ = 0.046, p = 0.83).

In a previous study, it was established that liver stage of merozoites double every 8 h. Hence, each hepatocyte contains thousands of merozoites [16]. Moreover, in early stage infections in non- or partially immune patients, such as the children included in this study, parasite multiplication during intra-erythrocytic stage range from six to tenfold per cycle, and in some cases up to 20-fold has been reported [17, 18]. It takes 48 h for *P. falciparum* to complete one cycle. Within 3–4 cycles, total falciparum parasitaemia increase exponentially from one parasite to 10^8^ (thus 100 million) parasites, an increase of over about 520,000 parasites each hour which corresponds to an increase of 50 parasites/μL hourly [19]. This level of parasitaemia is usually associated with the onset of parasite-induced fever and malaria-related symptoms in nonimmune individuals [20]. It could therefore be speculated that malaria parasites were released slowly into peripheral blood. The most probable reason underpinning this speculation was that submicroscopic parasitaemia may exist in all the patients that were previously negative by microscopy but later tested positive. This was because malaria antigens were detected in all of them. Rapidly dividing pre-erythrocytic parasites together with multiplying intra-erythrocytic parasites increased parasitaemia in an exponential manner.

After the 12 h microscopy testing procedure, 103 patients with suspected malaria remained smear negative for *Plasmodium* spp. The mean temperature in these patients was 37.6 °C, and it is possible that subsequent parasitological evaluation may have yielded further positive result. Furthermore, RDT yields improved with serial testing. These findings reveal that a single microscopic or RDT test for malaria reporting underestimates detection by 11.9% and 4.6% respectively.

Not every pyrexia is due to malaria. Viral, bacterial, mycobacterial, fungal, other parasitic infections, systemic inflammatory conditions and malignancies are important alternative diagnoses in children that present with fever. It is recommended that clinical and laboratory evaluation of pyrexia must be done exhaustively to unearth the actual origin of pyrexia.

## Conclusion

This study reports that one-time testing for malaria parasites underestimates malaria diagnosis by 12%. We recommend that children showing classical symptoms of malaria, but with initial negative parasitological reports be retested at 6 and 12 h to confirm or exclude the diagnosis of malaria.

### Limitation

In order not to unduly keep the pyretic children in the health facility, parasitological assessment was done up to 12 h.

## Data Availability

All study data collected in this study are presented in this publication.

## Abbreviations

µL: Microliter
LLIN: Long-lasting insecticide net
RDT: Rapid diagnostic test
WHO: World Health Organization

## References

[1] Centre for Disease Control and Prevention. Malaria. 2019; https://www.cdc.gov/dpdx/malaria/index.html. Assessed 14 August 2020.

[2] WHO. World malaria report 2019. Geneva: World Health Organization; 2019. https://www.who.int/news-room/feature-stories/detail/world-malaria-report-2019. Accessed 27 August 2020.

[3] World Health Organization. Malaria in children under five. 2018. https://www.who.int/malaria/areas/high_risk_groups/children/en/.

[4] Pasvol G (2005). Management of severe malaria: interventions and controversies. Infect Dis Clin North Am.

[5] WHO. Malaria key facts. 2018. https://www.who.int/news-room/fact-sheets/detail/malaria. Assessed 14 August 2020.

[6] Nandwani S, Mathur M, Rawat S (2005). Evaluation of the polymerase chain reaction analysis for diagnosis of falciparum malaria in Delhi India. Indian J Med Microbiol.

[7] Moody A (2002). Rapid diagnostic test for malaria parasites. Clin Microbiol.

[8] Wongsrichanalai C, Barcus MJ, Muth S, Sutamihardja A, Wernsdorfer WH (2007). A review of malaria diagnostic tools: microscopy and rapid diagnostic test (RDT). Am J Trop Med Hyg.

[9] Wu L, van den Hoogen LL, Slater H, Walker PG, Ghani AC, Drakeley CJ (2015). Comparison of diagnostics for the detection of asymptomatic *Plasmodium falciparum* infections to inform control and elimination strategies. Nature.

[10] Feleke DG, Tarko S, Hadush H (2017). Performance comparison of CareStartTM HRP”/pLDH combo rapid malaria test with light microscopy in north-western Tigray, Ethiopia: a cross-sectional study. BMC Infect Dis.

[11] Batwala V, Magnussen P, Nuwaha F (2010). Are rapid diagnostic tests more accurate in diagnosis of *Plasmodium falciparum* malaria compared to microscopy at rural health centres?. Malar J.

[12] WHO, Basic Malaria Microscopy: Part I Learner’s Guide, World Health Organization, Geneva, Switzerland, 1991.

[13] Amoah LE, Abankwa J, Oppong A (2016). *Plasmodium falciparum* histidine rich protein-2 diversity and the implications for PfHRP 2: based malaria rapid diagnostic tests in Ghana. Malar J.

[14] Agaba BB, Yeka A, Nsobya S (2019). Systematic review of the status of *pfhrp2* and *pfhrp3* gene deletion, approaches and methods used for its estimation and reporting in *Plasmodium falciparum* populations in Africa: review of published studies 2010–2019. Malar J..

[15] Aluka TM, Gyuse AN, Udonwa NE (2013). Comparison of cold water sponging and acetaminophen in control of fever among children attending a tertiary hospital in South Nigeria. J Fam Med Prim Care.

[16] White NJ, Pukrittayakamee S, Hien TT, Faiz MA, Mokuolu OA, Dondorp AM (2014). Malar Lancet.

[17] Simpson JA, Aarons L, Collins WE, Jeffery GM, White NJ (2002). Population dynamics of untreated *Plasmodium falciparum* malaria within the adult human host during the expansion phase of the infection. Parasitology.

[18] Dietz K, Raddatz G, Molineaux L (2006). Mathematical model of the first wave of *Plasmodium falciparum* asexual parasitaemia in nonimmune and vaccinated individuals. Am J Trop Med Hyg.

[19] White NJ (2002). The assessment of antimalarial drug efficacy. Trends Parasitol.

[20] Trampuz A, Jereb M, Muzlovic I, Prabhu M (2003). Clinical review: severe malaria. Crit Care.

